# Acute phase responses in clinically healthy multiparous Holsteins with and without calcium dysregulation during the early postpartum period

**DOI:** 10.3168/jds.2024-25300

**Published:** 2024-11-08

**Authors:** J. A. Seminara, C. R. Seely, J. A. A. McArt

**Affiliations:** 1 Department of Population Medicine and Diagnostic Sciences, College of Veterinary Medicine, Cornell University, Ithaca, NY 14850; 2 Department of Agriculture, Nutrition, and Food Systems, College of Life Sciences and Agriculture, University of New Hampshire, Durham, NH 03824

**Keywords:** dyscalcemia, inflammation, serum amyloid A, haptoglobin, lipopolysaccharide binding protein

## Abstract

Patterns of calcium dysregulation resulting in low total serum calcium concentrations (tCa) at 4 DIM, known as dyscalcemia, commonly occur in multiparous Holsteins. Dyscalcemia is associated with risk of disease, decreased production, and poor reproductive performance. Inflammation is well-documented early in lactation and is associated with similarly suboptimal outcomes. The acute phase response produces markers and mediators of inflammation; therefore, the objective of our case-control study was to evaluate postpartum patterns of 3 positive acute phase proteins in cows with and without dyscalcemia. We hypothesized that dyscalcemic cows would experience more activated inflammation than eucalcemic cows and that inflammation would precede dyscalcemia diagnosis. Multiparous Holstein cows at 2 commercial dairy farms in central New York were enrolled from a parent study based on tCa at 4 DIM, at a 2:1 ratio of eucalcemic (tCa >2.3 mmol/L; n = 32) to dyscalcemic cows (tCa <2.2 mmol/L; n = 16). Blood was collected 1 to 3 d before parturition and once every 24 h postpartum through 4 DIM. Samples were analyzed for 3 acute phase proteins, serum amyloid A (SAA), haptoglobin and LPS binding protein (LBP). Patterns of protein concentrations in blood over time were compared using linear mixed effects models including fixed effects of calcium status group, time, parity group, farm, relevant 2-way interactions, and the random effect of cow. Overall, dynamics of acute phase proteins showed that dyscalcemic cows experienced increased acute phase responses compared with eucalcemic cows, and that these responses preceded dyscalcemia diagnosis at 4 DIM. Dyscalcemic cows had elevated concentrations of SAA beginning at 2 DIM (eucalcemic: mean = 13.88 μg/mL, 95% CI = 11.34 to 16.99 μg/mL; dyscalcemic: mean = 32.95 μg/mL, 95% CI = 24.55 to 44.21 μg/mL) and continuing through 4 DIM (eucalcemic: mean = 8.14 μg/mL, 95% CI = 6.66 to 9.95 μg/mL; dyscalcemic: mean = 30.01 μg/mL, 95% CI = 22.60 to 39.83 μg/mL). Haptoglobin concentrations were also elevated in the blood of dyscalcemic cows from 2 DIM (eucalcemic: mean = 0.39 g/L, 95% CI = 0.31 to 0.49 g/L; dyscalcemic: mean = 1.11 g/L, 95% CI = 0.79 to 1.56 g/L) through 4 DIM (eucalcemic: mean = 0.27 g/L, 95% CI = 0.21 to 0.34 g/L; dyscalcemic: mean = 1.65 g/L, 95% CI = 1.19 to 2.28 g/L). Concentrations of LBP exhibited a different pattern with a small difference between groups at 3 DIM (eucalcemic: mean = 4.67 μg/mL, 95% CI = 4.02 to 5.42 μg/mL; dyscalcemic: mean = 7.91 μg/mL, 95% CI = 6.49 to 9.63 μg/mL) that became larger at 4 DIM (eucalcemic: mean = 4.88 μg/mL, 95% CI = 4.22 to 5.64 μg/mL; dyscalcemic: mean = 10.79 μg/mL, 95% CI = 8.84 to 13.17 μg/mL). Our work supports the hypothesis that dyscalcemia and inflammatory activity are associated in dairy cows under naturally occurring postpartum conditions. Although the causal structure of this relationship remains unknown, improved understanding of inflammation and dyscalcemia may provide insight into mechanisms by which some cows experience maladaptation during the early postpartum period.

## INTRODUCTION

Multiparous dairy cows commonly experience calcium dysregulation in the early postpartum period (Couto Serrenho et al., 2021; McArt and Oetzel, 2023). Also known as dyscalcemia, this condition is characterized by low total serum calcium concentrations (**tCa**) at 4 DIM (tCa ≤2.2 mmol/L; Neves et al., 2018; McArt and Neves, 2020), and has considerable incidence on commercial dairy farms, ranging from 21% to 72% (Mahjoubi et al., 2023; Seminara et al., 2023). Through a variety of epidemiological studies, dyscalcemia has been associated with increased risk of disease, decreased feed intake, poor production outcomes, and depressed reproductive efficiency (McArt and Neves, 2020; Seely et al., 2021; Seely and McArt, 2023a). Though the causal structures underlying these relationships have yet to be fully established, for now it is clear that dyscalcemia is an important indicator that cows may be struggling to adapt to the challenges of the early postpartum period.

Among numerous early lactation challenges, systemic inflammation is thought to be a central driver of lactational maladaptation (Sordillo and Raphael, 2013; Bradford and Swartz, 2020; Mezzetti et al., 2020). All cows experience some degree of inflammation following parturition, which is likely a normal response to restore tissue homeostasis in the reproductive tract (Humblet et al., 2006; Bionaz et al., 2007; Medzhitov, 2008). When systemic inflammation becomes excessive, however, it is associated with the onset of early lactation diseases as well as decreased reproductive success and reduced daily milk production (Huzzey et al., 2009; Nightingale et al., 2015; Martins et al., 2021). Evidence suggests that inflammation does not share these negative outcomes with dyscalcemia by chance. In a model of extreme induced inflammation, many species, including cows, experience a precipitous decline in blood calcium concentrations immediately following intravenous LPS challenge (Carlstedt et al., 2000; Toribio et al., 2005; Chandler et al., 2022). Taken with the knowledge of ubiquitous postpartum inflammation, this evidence hints at an association between inflammation and dyscalcemia in the early postpartum period.

Inflammation is often quantified in postpartum dairy cows via the acute phase response, an inflammatory process mediated by the liver during which hepatocytes shift their metabolic activity toward the production of positive acute phase proteins such as serum amyloid A (SAA), haptoglobin, and LPS binding protein (LBP; Humblet et al., 2006; Ceciliani et al., 2012; Trevisi and Minuti, 2018). Therefore, our objective in this exploratory study was to assess differences in the acute phase response between cows with and without dyscalcemia. We hypothesized that dyscalcemic cows would experience more extreme inflammatory activation than eucalcemic cows, marked specifically by increased serum concentrations of the acute phase proteins SAA, haptoglobin, and LBP. Further, we hypothesized that heightened inflammation would be begin before dyscalcemia diagnosis.

## MATERIALS AND METHODS

Our case-control study was designed and analyzed following the STROBE-Vet reporting guidelines (Sargeant et al., 2016).

### Sample Size Calculation

Our target sample size was established based on the intention to detect a difference in the blood concentrations of acute phase proteins between cows with and without dyscalcemia. We conducted a sample size calculation for each of our outcomes of interest, SAA, LBP, and haptoglobin, separately to ensure adequate enrollment for the assessment of each outcome. Each of the following calculations was performed controlling for type 1 error at 5%, and with power set at 90% to reduce the probability of type 2 error.

In the absence of data directly related to our goal, we used SAA and LBP results from Horst et al. (2020), which explored differences in these markers between cows with dysregulated and normally regulated calcium status in an LPS-challenge model. Assuming a mean blood SAA concentration of 600 μg/mL for dysregulated (i.e., dyscalcemic) cows and 385 μg/mL for normally regulated (i.e., eucalcemic) cows, with a SD of 244 μg/mL, we calculated a required sample size of 29 cows per group. Assuming mean LBP concentrations of 25 and 13.5 μg/mL for dysregulated cows and normally regulated cows, respectively, with a SD of 6.1 μg/mL, we arrived at a target sample size of 8 cows per group. To calculate the target sample size for haptoglobin, we used data from Huzzey et al. (2009), which found that cows with mild metritis had elevated concentrations of haptoglobin at 3 DIM compared with healthy cows. Based on the understanding that cows with calcium dysregulation are at higher risk of metritis (Neves et al., 2018), we assumed that dyscalcemic cows would have similar concentrations of haptoglobin to mildly metritic cows. Using the mean blood haptoglobin concentrations for metritic cows, 1.15 g/L, and for healthy cows, 0.50 g/L, with a SD of 0.72 g/L we arrived at a target sample size of 26 cows per group to detect a difference in haptoglobin concentrations. From these calculations, we selected 29 cows per group as the final target sample size to satisfy the requirements of assessing all selected acute phase proteins.

### Study Population and Sample Collection

Our study was conducted using banked samples from a previous field trial (Seely and McArt, 2023b). Briefly, the trial enrolled 89 multiparous Holsteins from 2 central New York farms that milked at least 1,000 Holstein cows 3 times per day, housed cows in freestall barns, fed a negative DCAD diet prepartum and a TMR postpartum, and did not apply blanket calcium supplementation to fresh cows. Blood was collected before feed delivery, from coccygeal vessels, once 1 to 3 d before parturition and once daily for the first 4 DIM. Serum was separated following clotting and centrifugation, aliquoted, and stored at −20°C until analysis.

Multiparous cows were retrospectively enrolled into the present study based on strict eligibility criteria. Cows were not eligible for enrollment if they received calcium therapy before 4 DIM, developed metritis or displaced abomasum during the first week of lactation, or were removed from the herd before 10 DIM. Health screening protocols were conducted by farm personnel and disease events were recorded following diagnosis by physical exam based on standard disease definitions. Cows experiencing rectal temperatures ≥39.5°C and also having red to brown uterine discharge were diagnosed with metritis. If the classical “ping” sound was detected during simultaneous auscultation and percussion on the left side of the cow, along the line between the tuber coxae and the olecranon, cows were diagnosed with a displaced abomasum. All diagnoses were subsequently confirmed by the herd veterinarian. Dyscalcemia diagnosis is commonly made at 4 DIM where tCa ≤2.2 mmol/L, a threshold calcium concentration that is associated with early lactation disease (McArt and Neves, 2020). This threshold has been shown to have a sensitivity of 76% and specificity of 80%, when used to predict adverse health events (metritis, displaced abomasum, hyperketonemia, and culling at <13 DIM) in early lactation multiparous Holsteins (Seminara et al., 2023). In an effort to improve the accuracy of our diagnoses and phenotypic characterization, cows were eligible for inclusion only if their calcium status at 4 DIM was unambiguous. Cows considered unambiguous with respect to calcium status were those firmly below the common tCa threshold for dyscalcemia and those above an elevated tCa threshold for eucalcemia (dyscalcemia: tCa <2.2. mmol/L; eucalcemia: tCa >2.3 mmol/L). These measures increased both the positive and negative predictive values of our diagnoses, thus making it more likely that the cows enrolled in our study truly experienced the phenotypes that we diagnosed. Given our target sample size of 29 cows per group, we enrolled all eligible dyscalcemic cows as cases (n = 16). Eucalcemic cows were selected randomly from the banked eucalcemic population such that the final proportion of eucalcemic cows to dyscalcemic cows would be 2:1 (n = 32).

### Laboratory Analysis

Serum samples were analyzed for SAA using a commercially available kit at the New York State Animal Health Diagnostic Center (Ithaca, NY) on an automated analyzer (Hitachi Modular P800, Roche Diagnostics). A second aliquot of serum was analyzed for haptoglobin at the Animal Health Laboratory (Guelph, Ontario, Canada) using a method based on Makimura and Suzuki (1982) on an automated analyzer (Cobas c501, Roche Diagnostics). A third aliquot was analyzed for LBP concentrations using commercially available ELISA kits (HK503, Hycult Biotech). Inter- and intra-assay CV were 4.0% and 0.03%, respectively, for SAA, 8.2% and 2.0%, respectively, for haptoglobin, and 12.5% and 5.4% respectively, for LBP.

### Statistical Analysis

All calculations of descriptive statistics and statistical analyses were performed using R version 4.4.0 in RStudio (Posit Software, PBC, Boston, MA). Descriptive statistics were generated using general functions contained in the base package of R. Statistical significance was declared at *P* < 0.05.

To assess prepartum differences in serum concentrations of acute phase proteins between calcium status groups, we used linear regression methods. Our model building procedure for each acute phase protein (SAA, haptoglobin, and LBP) was identical. A maximal model was fit to predict each acute phase protein concentration that included Ca status group (eucalcemic or dyscalcemic), parity group (2, 3, and ≥4), and farm (A or B), as well as all 2-way interactions as fixed effects in the model. The 2-way interaction terms offered to the prepartum models were Ca status group × parity group, Ca status group × farm, and farm × parity group. A backward stepwise elimination process was used to remove interaction terms with *P* > 0.05. The covariate of interest, Ca status group, was retained in every model, as were farm and parity, due to their known confounding effects at the population level.

To evaluate postpartum differences in acute phase protein concentrations over time we used the nlme package to fit a linear mixed effects model for each acute phase protein. The same model building procedure noted above was implemented with the addition of time and its interactions with all other covariates in the maximal model. Calcium status group, time, and their interaction term were included in models regardless of statistical significance, as the variables of primary interest. Models also incorporated parity group and farm even when *P* > 0.05, as both factors confound at the population level. The random effect of cow was included to account for intracow correlation between samples taken from the same individual at multiple time points. Models were tested with 3 correlation structures: autoregressive, compound symmetry, and unstructured. The unstructured correlation was selected for SAA and haptoglobin as it resulted in the lowest Akaike information criteria for these acute phase proteins. Unstructured correlation was also selected for LBP despite its Akaike information criterion being roughly equivalent to that of the autoregressive structure because unstructured correlation requires the fewest assumptions about the underlying data.

For both pre- and postpartum models, model fit was assessed through visual inspection of diagnostic plots. Residuals were plotted over the range of predicted values to test the assumption of homoscedasticity and histograms were used to evaluate normality of residuals. Concentrations of each acute phase protein were log-transformed in all models to improve homoscedasticity and normality of residuals, which were compromised in nontransformed models. We calculated proportionally weighted marginal means for each calcium status group at each day from each model and conducted pairwise comparisons at each day using the emmeans package, which adjusts for multiple comparisons using the Tukey method. The same procedure was used to calculate proportional means of covariates retained in the final model with *P* < 0.05. Results are presented as back-transformed marginal means with 95% CI calculated from SE.

## RESULTS

### Descriptive Characteristics

Of the 89 cows in the original cohort study from which we sampled, 16 dyscalcemic cows were eligible for enrollment in our study (farm A, n = 9; farm B, n = 7). Dyscalcemic cows were matched with 32 eucalcemic cows from the original cohort, following control selection criteria outlined above (farm A, n = 13; farm B, n = 19), resulting in a total study sample size of 48 cows. Average daily milk yield through 10 wk in milk was 47.5 kg/d for farm A and 49.1 kg/d for farm B. Of the eucalcemic cows, 12 were parity 2, 15 were parity 3, and 5 were parity ≥4; the dyscalcemic group included 6 for parity 2, 5 for parity 3, and 5 for parity ≥4 cows (Table 1). Diet information and data concerning disease incidence after the first week of lactation are detailed elsewhere (Seely and McArt, 2023b). The means and SD of total serum calcium and each of the measured acute phase proteins, at each time point, for eucalcemic and dyscalcemic cows are contained in Table 2.

### Acute Phase Proteins

#### Serum Amyloid A.

**Figure 1.**
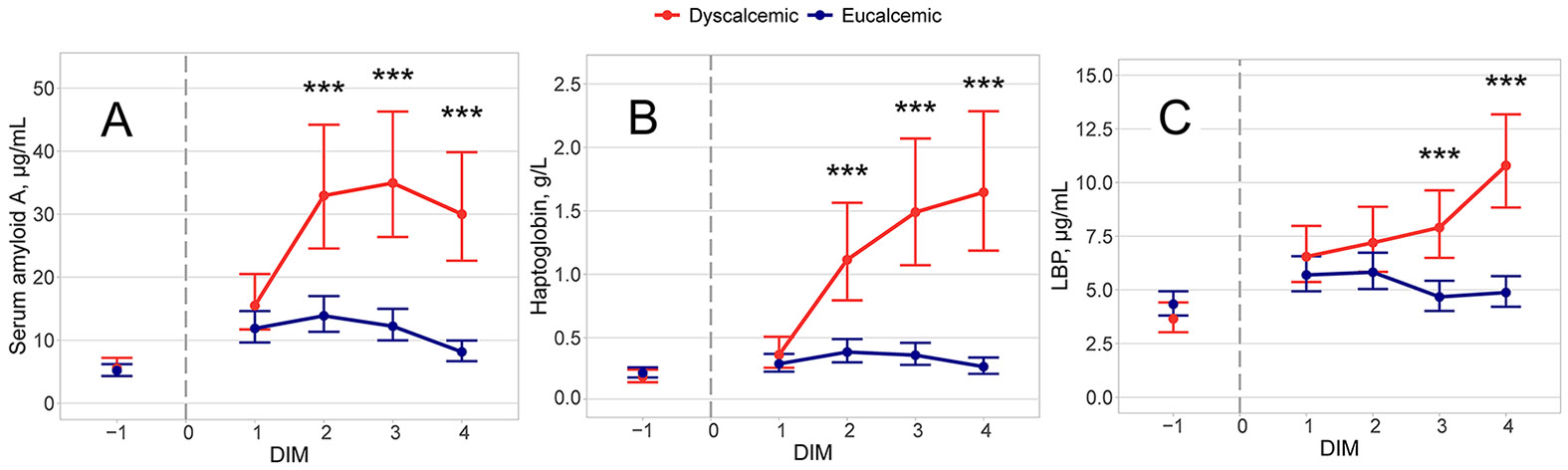
Modeled and back-transformed concentrations of SAA (A), haptoglobin (B), and LBP (C) at 1 d before parturition and from 1 through 4 DIM by Ca status group, for 48 multiparous Holsteins on 2 commercial dairy farms in central New York. Calcium status group classification was based on serum tCa at 4 DIM (dyscalcemic: tCa <2.2 mmol/L [n = 16]; eucalcemic: tCa >2.3 mmol/L [n = 32]). Asterisks (***) indicate differences between groups at *P* < 0.001, and error bars represent 95% CI calculated from SEM.

Concentrations of SAA over time by calcium status group are displayed in Figure 1A. Prepartum, we observed no difference in SAA concentrations between calcium status groups (eucalcemic: mean = 5.16 μg/mL, 95% CI = 4.30 to 6.19 μg/mL; dyscalcemic: mean = 5.55 μg/mL, 95% CI = 4.28 to 7.19 μg/mL; *P* = 0.7), when controlling for parity (*P* = 0.5); however, we did detect a difference between farms (farm A: mean = 6.54 μg/mL, 95% CI = 5.24 to 8.16 μg/mL; farm B: mean = 4.41 μg/mL, 95% CI = 3.60 to 5.40 μg/mL; *P* = 0.01). The final prepartum model had df = 43.

The final postpartum model for SAA included the variables Ca status group (*P* < 0.001), time (*P* < 0.001), Ca status group × time (*P* < 0.001), parity group (*P* = 0.3), parity group × time (*P* = 0.03), and farm (*P* = 0.8), with df = 25. Patterns of postpartum SAA concentrations over time differed by calcium status group such that we observed no difference in SAA concentrations between calcium status groups at 1 DIM (eucalcemic: mean = 11.87 μg/mL, 95% CI = 9.64 to 14.62 μg/mL; dyscalcemic: mean = 15.49 μg/mL, 95% CI = 11.71 to 20.49 μg/mL; *P* = 0.1) but at 2 DIM the SAA concentrations of dyscalcemic cows increased above those of eucalcemic cows (eucalcemic: mean = 13.88 μg/mL, 95% CI = 11.34 to 16.99 μg/mL; dyscalcemic: mean = 32.95 μg/mL, 95% CI = 24.55 to 44.21 μg/mL; *P* < 0.001). Concentrations of SAA in the blood of dyscalcemic cows remained higher than those of eucalcemic cows at both 3 DIM (eucalcemic: mean = 12.22 μg/mL, 95% CI = 9.97 to 14.99 μg/mL; dyscalcemic: mean = 34.94 μg/mL, 95% CI = 26.38 to 46.28 μg/mL; *P* < 0.001), and 4 DIM (eucalcemic: mean = 8.14 μg/mL, 95% CI = 6.66 to 9.95 μg/mL; dyscalcemic: mean = 30.01 μg/mL, 95% CI = 22.60 to 39.83 μg/mL; *P* < 0.001). The parity group × time interaction revealed a difference in SAA patterns by parity group whereby cows with parity ≥4 had lower concentrations of SAA than parity 2 cows at 1 DIM (parity 2: 16.85 μg/mL, 95% CI = 12.73 to 22.29 μg/mL; parity ≥4: mean = 9.35 μg/mL, 95% CI = 6.55 to 13.35 μg/mL; *P* = 0.03).

#### Haptoglobin.

Concentrations of haptoglobin over time by calcium status group are displayed in Figure 1B. When controlling for parity (*P* = 0.8), prepartum haptoglobin concentrations were not different between calcium status groups (eucalcemic: mean = 0.22 g/L, 95% CI = 0.19 to 0.27 g/L; dyscalcemic: mean = 0.19 g/L, 95% CI = 0.15 to 0.25 g/L; *P* = 0.4), but we did detect a difference between farms (farm A = 0.26 g/L, 95% CI = 0.21 to 0.32 g/L; farm B = 0.17 g/L, 95% CI = 0.14 to 0.21 g/L; *P* = 0.008). The final prepartum model had df = 34.

Postpartum, the final model for haptoglobin included the variables Ca status group (*P* < 0.001), time (*P* < 0.001), Ca status group × time (*P* < 0.001), parity group (*P* = 0.3), and farm (*P* = 0.004), with df = 19. Haptoglobin concentrations, when controlled for both parity and farm, exhibited temporal changes that differed by calcium status group. Though eucalcemic and dyscalcemic cows had comparable haptoglobin concentrations at 1 DIM (eucalcemic: mean = 0.29 g/L, 95% CI = 0.23 to 0.37 g/L; dyscalcemic: mean = 0.37 g/L, 95% CI = 0.26 to 0.51 g/L; *P* = 0.3), cows with dyscalcemia experienced an increase in haptoglobin concentrations at 2 DIM whereas haptoglobin concentrations in the blood of eucalcemic cows remained low (eucalcemic: mean = 0.39 g/L, 95% CI = 0.31 to 0.49 g/L; dyscalcemic: mean = 1.11 g/L, 95% CI = 0.79 to 1.56 g/L; *P* < 0.001). Eucalcemic cows maintained low concentrations of haptoglobin in their blood for the remainder of the sampling period, whereas haptoglobin concentrations in the blood of dyscalcemic cows continued to rise at 3 DIM (eucalcemic: mean = 0.36 g/L, 95% CI = 0.29 to 0.46 g/L; dyscalcemic: mean = 1.49 g/L, 95% CI = 1.07 to 2.07 g/L; *P* < 0.001) and 4 DIM (eucalcemic: mean = 0.27 g/L, 95% CI = 0.21 to 0.34 g/L; dyscalcemic: mean = 1.65 g/L, 95% CI = 1.19 to 2.28 g/L; *P* < 0.001). The effect of farm in our model revealed that on average cows on farm A had higher concentrations of haptoglobin than those on farm B (farm A: mean = 0.59 g/L, 95% CI = 0.48 to 0.72 g/L; farm B: mean = 0.40 g/L, 95% CI = 0.33 to 0.49 g/L; *P* = 0.006).

#### Lipopolysaccharide Binding Protein.

Concentrations of LBP over time by calcium status group are displayed in Figure 1C. Again, we found no prepartum difference between calcium status groups (eucalcemic: mean = 4.34 μg/mL, 95% CI = 3.81 to 4.93 μg/mL; dyscalcemic: mean = 3.66 μg/mL, 95% CI = 3.03 to 4.42 μg/mL; *P* = 0.1), although we observed a pronounced difference by farm (farm A: mean = 5.23 μg/mL, 95% CI = 4.48 to 6.11 μg/mL; farm B = 3.29 μg/mL, 95% CI = 2.84 to 3.82 μg/mL; *P* < 0.001). The prepartum model had df = 35 and we did not detect a parity effect (*P* = 0.8).

Following parturition, the final model for LBP included the variables Ca status group (*P* < 0.001), time (*P* = 0.1), Ca status group × time (*P* < 0.001), parity group (*P* = 0.7), and farm (*P* = 0.2), with df = 19. Concentrations of LBP displayed changes that were distinct between calcium status groups over time, when controlling for parity and farm. These patterns revealed that calcium status groups had similar concentrations of LBP at both 1 DIM (eucalcemic: mean = 5.69 μg/mL, 95% CI = 4.93 to 6.57 μg/mL; dyscalcemic: mean = 6.55 μg/mL, 95% CI = 5.37 to 7.98 μg/mL; *P* = 0.3) and 2 DIM (eucalcemic: mean = 5.82 μg/mL, 95% CI = 5.03 to 6.73 μg/mL; dyscalcemic: mean = 7.20 μg/mL, 95% CI = 5.84 to 8.87 μg/mL; *P* = 0.1). Although eucalcemic cows maintained low blood concentrations of LBP, dyscalcemic cows experienced an elevation in LBP concentrations at 3 DIM (eucalcemic: mean = 4.67 μg/mL, 95% CI = 4.02 to 5.42 μg/mL; dyscalcemic: mean = 7.91 μg/mL, 95% CI = 6.49 to 9.63 μg/mL; *P* < 0.001), and an even greater rise in LBP concentrations at 4 DIM (eucalcemic: mean = 4.88 μg/mL, 95% CI = 4.22 to 5.64 μg/mL; dyscalcemic: mean = 10.79 μg/mL, 95% CI = 8.84 to 13.17 μg/mL; *P* < 0.001).

## DISCUSSION

Our objective was to determine whether postpartum acute phase responses differed between multiparous Holstein cows with and without calcium dysregulation. We found that serum concentrations of the acute phase proteins SAA, haptoglobin, and LBP all increased within the first 4 d after parturition in cows developing dyscalcemia but not in eucalcemic cows. This evidence strongly supports the hypothesis that the acute phase response is activated to a greater extent in cows that subsequently experience calcium dysregulation and suggests that inflammatory activation and dyscalcemia may be interrelated processes under naturally occurring postpartum conditions.

The minimal inflammatory activation we observed in eucalcemic cows across our analytes was a defining and unexpected feature of our data. Although the pattern of normal postpartum LBP concentrations is not well-defined, it is commonly accepted that in the first week postpartum even healthy cows experience elevated concentrations of SAA and haptoglobin (Humblet et al., 2006; Chan et al., 2010; Brodzki et al., 2015). Our data generally supported this hypothesis, as our study was conducted on clinically healthy cows, and we detected significant increases over time in the concentrations of both analytes. When stratified by calcium status, however, we found that eucalcemic cows had only marginally active acute phase responses during the early postpartum period. It is possible that postpartum inflammation may be mediated by mechanisms outside of the acute phase response under eucalcemic conditions and may require assessment using other markers of inflammatory status. Our data are a signal that calcium status should be considered when evaluating the hypothesis of ubiquitous postpartum inflammation.

Well-conserved across vertebrate species, SAA is generally considered the prototypical positive acute phase protein (Uhlar and Whitehead, 1999). In response to acute inflammatory stimuli, such as tissue injury or infection, the liver can quickly produce tremendous amounts of SAA reaching blood concentrations 1,000-fold greater than its baseline in 24 h (Sack, 2018). The peak SAA concentration attained by dyscalcemic cows in our study was only 35.64 μg/mL, a mere 6-fold increase over the prepartum baseline. Some groups have reported SAA concentrations greater than 75 μg/mL in the first week of lactation, suggesting that the dyscalcemic cows enrolled in our study experienced a subdued SAA response (Humblet et al., 2006; Couto Serrenho et al., 2023). Though this is possible, it is quite difficult to compare SAA concentrations across studies because of large intercow variability in SAA responses, differences in farm-level factors known to influence SAA concentrations, and inconsistent sample storage times contributing to reductions in measurable SAA (Jacobsen et al., 2004; Tóthová et al., 2012; Schmitt et al., 2021). Additionally, it is not unheard of for clinically healthy cows in the early postpartum to have low SAA concentrations on commercial dairy farms. Schmitt et al. (2021) sampled clinically healthy cows once during the first week of lactation on 10 farms and found that the average concentration of SAA was less than 40 μg/mL at 6 out of 10 of these farms. In this context, the strong statistical association we observed between elevated SAA concentrations and dyscalcemia on 2 different dairy farms may be more biologically relevant than the magnitude of the SAA increase.

Haptoglobin is arguably the most well studied bovine positive acute phase protein, and unlike SAA, it did reach extreme concentrations in the blood of dyscalcemic cows. Haptoglobin tends respond 48 h after the initial insult and resolves more slowly than SAA (Ceciliani et al., 2012; Chandler et al., 2022). We observed a robust haptoglobin response among dyscalcemic cows at 48 h postpartum suggesting that the inflammatory event stimulating haptoglobin production in dyscalcemic cows is taking place at the time of parturition. Elevated haptoglobin concentrations are associated with many types of inflammatory processes in the dairy cow, making haptoglobin a useful but somewhat nonspecific marker of inflammation (Alsemgeest et al., 1994). During the early postpartum period in specific, high concentrations of haptoglobin have been consistently associated with reproductive disease and dysfunction (Huzzey et al., 2009; Dubuc et al., 2010; Nightingale et al., 2015). The concentrations of haptoglobin that we observed in the blood of dyscalcemic cows were comparable to previously reported values for cows experiencing metritis (Burfeind et al., 2014). Cows with metritis, induce robust haptoglobin responses by 48 h postpartum and have depressed blood Ca status by 4 DIM (Martinez et al., 2012; Venjakob et al., 2021; Menta et al., 2023). Combined, this evidence could suggest that dyscalcemic cows in our study may have experienced some level of underlying uterine inflammation which did not progress to clinical disease.

At first glance, our SAA results are incongruous with this hypothesis, because after the first week of lactation SAA has been used to predict inflammation in the reproductive tract with good success (sensitivity = 0.95, specificity = 0.67; Kaya et al., 2016). However, within the first week of lactation, published data do not show a strong relationship between SAA and reproductive disease, suggesting that even metritic cows may have blood concentrations of SAA corresponding to healthy animals at this time (Chan et al., 2010; Brodzki et al., 2015; Schmitt et al., 2021). Although, we cannot say definitively whether underlying uterine inflammation was the main driver of the acute phase response we observed, our data seems consistent with this hypothesis. It is also possible that cows in our study experienced undetected metritis. Due to inconsistent disease definitions, diagnostic approaches, and timing of diagnoses, metritis detection often has variability on dairy farms (Sannmann et al., 2012; Espadamala et al., 2016). Though previous research has already established associations at the intersection of reproductive health, systemic inflammation and calcium dysregulation (Martinez et al., 2012; Venjakob et al., 2019), this remains a critical area for further future study.

From the temporal dynamics of SAA and haptoglobin, it is apparent that the acute phase response becomes activated in dyscalcemic cows by 48 h and continues to be highly active through 96 h postpartum. Though LBP in dyscalcemic cows did eventually rise to concentrations consistent with elevated inflammation (Opgenorth et al., 2024), the temporal dynamics of this acute phase protein were distinct from the other two. We observed a progressive increase in LBP over time, whereby concentrations were mildly increased by 3 DIM and considerably elevated at 4 DIM postpartum. This dynamic pattern is suggestive of underlying pathophysiology. Lipopolysaccharide binding protein is essential in the innate immune response against bacteria and is most well-known for its role in the neutralization and clearance of LPS (Wurfel et al., 1994; Gutsmann et al., 2001; Ceciliani et al., 2012). In bovine LPS-challenge models, LBP is induced within 24 h, indicating that the regulatory pathway controlling LBP secretion is highly responsive to blood concentrations of LPS (Al-Qaisi et al., 2020; Horst et al., 2020; Opgenorth et al., 2024). Though we can only speculate about the reasons for LBP concentrations becoming elevated in our study, the gradual pattern of increasing LBP is not consistent with high levels of LPS entering the blood in a single event, such as parturition or any other time in our sample period. Instead, the activation pattern of LBP concentrations would suggest a gradual and progressive introduction of LPS with a continuous source. One potential continuous source of LPS could be the gut. As cows transition from a low energy dry diet to a higher energy early lactation diet, the gut epithelium is thought to lose some of its barrier functions allowing LPS to enter the bloodstream (Mezzetti et al., 2021). Although this might explain a gradual rise in LBP, it is not obvious why eucalcemic cows that have recently made the same dietary transition would not be similarly affected. Another potential source of LPS is the reproductive tract. By mechanisms that are not fully understood, LPS is absorbed from the uterus into the bloodstream in cows that develop metritis (Mateus et al., 2003; Magata et al., 2015, 2017). Magata et al. (2017) showed a progressive pattern of increasing LPS in the blood of cows with metritis over the first week of lactation, which matches with the progressive increase in LBP we observed in dyscalcemic cows. Based on these findings, a hypothesis of underlying subclinical uterine inflammation could explain each of the results we observed, but assessing reproductive disease was not an objective of this study, and therefore we can only guess at the true underlying causes of the acute phase response we observed.

Evidence from the LPS-challenge model was a key motivator for this study (Chandler et al., 2022; Opgenorth et al., 2024). Using this model, researchers have shown that extreme inflammatory activation leads to a depression in blood calcium concentrations. We hypothesized, based on that evidence, that inflammation could be related to postpartum calcium dysregulation under natural conditions, and the findings of this study have shown that a temporal association is present between inflammatory activation and dyscalcemia. However, the magnitude of response for each analyte and their specific temporal dynamics are widely divergent from research published using the LPS-challenge model. Combined, these data clearly show that the LPS-challenge model does not accurately recapitulate inflammatory insults associated with parturition; thus, conclusions drawn from the assumption that it does, should be interpreted carefully and with reservation.

An important limitation of our study was the case-control design. We did not include the full spectrum of calcium statuses within the population, as our study design aimed to ensure cows were truly dyscalcemic or eucalcemic. It is therefore possible that the association between inflammatory activation and dyscalcemia would not be as clear as we have shown, if cows with more intermediate calcium statuses were included in an analysis. Another limitation of the design was that this study was exploratory in nature; the parent study by Seely and McArt (2023b) was not designed to answer this question. This presented some potential statistical limitations, because our study population was not balanced by farm or parity. Though we cannot ignore the possibility that imbalance in our data could have been problematic, mixed effects models are generally robust to imbalance in repeated measures data and tend not to produce biased estimates in these contexts (Laird and Ware, 1982; Cnaan et al., 1997; Oberpriller et al., 2022). It is also worth noting that we did not reach our target sample size, but the association between dyscalcemia and inflammation was strong enough to reach statistical significance in spite of the small sample size.

Though it is impossible to draw a causal inference from the data we have presented, we show that under naturally occurring postpartum conditions, elevated inflammation and calcium dysregulation have a temporal association whereby inflammatory activation precedes dyscalcemia diagnosis at 4 DIM. Based on this evidence, it seems likely that whatever pattern of lactational maladaptation is driving dyscalcemia progression might be inducing inflammatory responses first. This yet undefined maladaptive process may cause both inflammation and dyscalcemia at the same time, but it is also quite possible that inflammation itself, caused by underlying maladaptation, is the main driver of dyscalcemia. Unraveling the biological mechanisms that tie these processes together is a critical direction for future research so that prevention methods and therapeutic strategies can be optimized to improve outcomes for dairy cows that have become maladapted in the early postpartum period.

## CONCLUSIONS

Clinically healthy cows that go on to experience postpartum calcium dysregulation experience increased acute phase responses in the immediate days following parturition compared with eucalcemic cows. This enhanced inflammatory activity begins before the diagnosis of dyscalcemia. These findings allude to some greater pattern of lactational maladaptation tying postpartum inflammation and dyscalcemia together, though the drivers of these phenomena remain obscure. The pathophysiological mechanisms underlying these relationships must be investigated further under naturally occurring postpartum conditions to develop therapeutic strategies that can mitigate the negative outcomes associated with postpartum maladaptation.

## Figures and Tables

**Table 1.** Descriptive characteristics of the study population composed of 48 multiparous Holsteins on 2 commercial dairy farms in central New York, by Ca status group, parity group, and farm

Farm	Ca status group^1^	Parity group		
		2	3	≥4
A	Eucalcemic	7	5	1
	Dyscalcemic	2	2	5
B	Eucalcemic	5	10	4
	Dyscalcemic	4	3	0

1 Calcium status group classification was based on tCa at 4 DIM (dyscalcemic: tCa <2.2 mmol/L [n = 16]; eucalcemic: tCa >2.3 mmol/L [n = 32]).

**Table 2.** Descriptive statistics (mean ± SD) of tCa, SAA, haptoglobin, and LBP, at 1 d before parturition and from 1 through 4 DIM by Ca status group, for 48 multiparous Holsteins on 2 commercial dairy farms in central New York

Item	Group^1^	DIM				
		−1	1	2	3	4
tCa, mmol/L	Eucalcemic	2.4 (±0.1)	1.9 (±0.3)	2.2 (±0.2)	2.3 (±0.1)	2.4 (±0.1)
	Dyscalcemic	2.4 (±0.1)	1.9 (±0.3)	1.9 (±0.3)	2.0 (±0.2)	2.1 (±0.1)
SAA, μg/mL	Eucalcemic	6.06 (±4.77)	13.93 (±6.42)	16.00 (±10.27)	17.21 (±15.94)	9.13 (±5.93)
	Dyscalcemic	6.69 (±4.35)	17.19 (±8.66)	37.77 (±20.45)	39.56 (±18.65)	34.33 (±20.40)
Haptoglobin, g/L	Eucalcemic	0.25 (±0.16)	0.34 (±0.24)	0.50 (±0.38)	0.55 (±0.57)	0.40 (±0.52)
	Dyscalcemic	0.22 (±0.14)	0.48 (±0.41)	1.25 (±0.44)	1.66 (±0.65)	2.00 (±1.13)
LBP, μg/mL	Eucalcemic	4.65 (±1.81)	5.77 (±1.21)	6.07 (±2.31)	5.27 (±3.52)	5.61 (±3.13)
	Dyscalcemic	3.90 (±1.25)	6.84 (±2.18)	7.54 (±2.23)	8.97 (±6.11)	11.43 (±3.80)

1 Calcium status group classification was based on tCa at 4 DIM (dyscalcemic: tCa <2.2 mmol/L [n = 16]; eucalcemic: tCa >2.3 mmol/L [n = 32]).

## Nonstandard abbreviations used

tCa: total serum calcium concentration
